# A Computational Biomechanics Human Body Model Coupling Finite Element and Multibody Segments for Assessment of Head/Brain Injuries in Car-To-Pedestrian Collisions

**DOI:** 10.3390/ijerph17020492

**Published:** 2020-01-13

**Authors:** Chao Yu, Fang Wang, Bingyu Wang, Guibing Li, Fan Li

**Affiliations:** 1 School of Mechanical and Automotive Engineering, Xiamen University of Technology, Xiamen 361024, China; yuchao19930704@gmail.com (C.Y.);; 2 Fujian Collaborative Innovation Center for R&D of Coach and Special Vehicle, Xiamen 361024, China; 3 School of Automotive and Mechanical Engineering, Changsha University of Science & Technology, Changsha 410205, China; 4 School of Mechanical Engineering, Hunan University of Science and Technology, Xiangtan 411201, China; 5 State Key Laboratory of Advanced Design and Manufacturing for Vehicle Body, Hunan University, Changsha 410082, China

**Keywords:** car-to-pedestrian collision, computational efficiency, coupling, finite element model, head/brain injury, multibody model

## Abstract

It has been challenging to efficiently and accurately reproduce pedestrian head/brain injury, which is one of the most important causes of pedestrian deaths in road traffic accidents, due to the limitations of existing pedestrian computational models, and the complexity of accidents. In this paper, a new coupled pedestrian computational biomechanics model (CPCBM) for head safety study is established via coupling two existing commercial pedestrian models. The head–neck complex of the CPCBM is from the Total Human Model for Safety (THUMS, Toyota Central R&D Laboratories, Nagakute, Japan) (Version 4.01) finite element model and the rest of the parts of the body are from the Netherlands Organisation for Applied Scientific Research (TNO, The Hague, The Netherlands) (Version 7.5) multibody model. The CPCBM was validated in terms of head kinematics and injury by reproducing three cadaveric tests published in the literature, and a correlation and analysis (CORA) objective rating tool was applied to evaluate the correlation of the related signals between the predictions using the CPCBM and the test results. The results show that the CPCBM head center of gravity (COG) trajectories in the impact direction (YOZ plane) strongly agree with the experimental results (CORA ratings: Y = 0.99 ± 0.01; Z = 0.98 ± 0.01); the head COG velocity with respect to the test vehicle correlates well with the test data (CORA ratings: 0.85 ± 0.05); however, the correlation of the acceleration is less strong (CORA ratings: 0.77 ± 0.06). No significant differences in the behavior in predicting the head kinematics and injuries of the tested subjects were observed between the TNO model and CPCBM. Furthermore, the application of the CPCBM leads to substantial reduction of the computation time cost in reproducing the pedestrian head tissue level injuries, compared to the full-scale finite element model, which suggests that the CPCBM could present an efficient tool for pedestrian brain-injury research.

## 1. Introduction

According to the latest report by the World Health Organization, about 1.25 million people worldwide die from road traffic accidents each year, of which pedestrians account for about 22% of the fatal injuries [1]. The traffic accident reconstruction technology has been widely utilized to analyze the cause of human injuries in the accident, further improve the safety design of vehicles [2], and eventually prevent or reduce the fatal damage to pedestrians. Human body computational biomechanics models are the core of traffic accident reconstruction technology, which have become one of the most important tools in the pedestrian protection research and automotive safety design.

At present, the pedestrian computational biomechanics models used in the study of pedestrian protection mainly include multibody (MB) (e.g., the Netherlands Organisation for Applied Scientific Research (TNO) model [3]) and finite element (FE) models (e.g., the Total Human Model for Safety (THUMS) model by Toyota Central R&D Laboratories [4,5]). The TNO model has been validated under various collision scenarios, by means of cadaver tests [6,7], dummy tests [2], and the pedestrian kinematics/injury prediction from the reconstruction of real-world accidents [8,9,10]. For instance, Linder et al. [9] reconstructed six car-to-pedestrian accidents using the TNO model, and the results showed that the impact locations of the vehicle exterior coincided with the reported vehicle damage, and the predicted pedestrian throw distance errors were within 20% when compared to the actual collision data. The THUMS model, as one of the most widely used pedestrian FE models, has been validated at both the segment [11,12,13] and the full-scale levels [14,15,16]. Recently, Wu et al. [17] further evaluated the biofidelity of the THUMS model (Version 4.01) in a whole-body car-to-pedestrian impact condition by reproducing three full-scale postmortem human subjects (PMHSs) impact tests, in which the whole body’s response data (that included head, thoracic T1 and T8, pelvis, left and right femur, etc.) between the simulation and experiment were evaluated.

As is well-known, the MB model is far less realistic than the FE model in terms of human biofidelity and the contacts definition between the rigid bodies, and is not able to calculate the human tissue level injury (such as stress, strain). However, such a model can efficiently reproduce the pedestrian kinematic in an accident and output the kinematic-based injuries, which is convenient for quick reconstruction of complicated traffic accidents or for massive simulations [18,19,20,21,22,23,24]. Compared to the MB model, the advantage of the FE model, being able to represent detailed human anatomical structure, makes it possible to study human tissue level injuries. However, the FE model requires high computational resources for the model adjustment and computations, which means that it is not realistic to conduct complicated traffic accident reconstruction using solely the FE model [25].

Head injury has been identified to be the most common cause of pedestrian fatality in pedestrian accidents, and substantial research efforts have been made focusing on pedestrian head injury biomechanics and prevention [25,26,27,28,29]. In this field, the FE model was frequently used for assessment of the pedestrian brain tissue injury, as the importance of brain deformation on traumatic brain injury had been extensively confirmed. However, due to the relatively low time efficiency in repositioning the impact conditions and simulating the impact of the accident event, the application of the full-scale FE model is now limited to parametric study concentrating on the defined impact scenarios by varying impact velocity, pedestrian position or angle with respect to the vehicle, e.g., [13,24,30]. In order to predict pedestrian head/brain injury responses in an accurate and efficient way by reproducing real-world car-to-pedestrian impact accidents, two popular approaches are usually employed:

(a) Firstly, multibody dynamics analysis software (such as MADYMO [31]) is used to reconstruct the accident, then the boundary conditions (e.g., initial linear and angular velocity/acceleration, initial position, etc.) at the instant of head–vehicle contact are extracted and applied to the isolated FE head model (head-only) complemented by the FE vehicle subsystem model (such as windshield or bonnet) to obtain skull fracture and brain tissue injury data [32,33,34,35].
(b) The accident is firstly reproduced with the full-scale MB models, similar to the above approach, and the time histories of the six degrees of freedom of the head center of gravity (COG) during the whole impact event are taken out from the MB reconstruction and prescribed to the head-only FE model to reproduce the brain tissue injury [36,37].

Both methods have dramatically decreased the time required for pedestrian repositioning and calculation compared to the full-scale FE models. However, there are still limitations. Specifically, the former one ignored the influence of the rest of the human body on head kinematics and injury response during the head–vehicle impact, which has been confirmed in the literature [38,39,40]. In addition, the head–ground impact was not considered; the latter one, relying on the assumption that the skull is a rigid body, may produce a remarkable difference in predicting brain tissue responses.

Overall, the obvious advantages and disadvantages of the FE and MB pedestrian computational biomechanics models mean that research on brain injury in car impact accidents is still challenging. In order to overcome these limitations. Shu [41] applied mesh morphing technology [42,43] to the study of impact injury in obese children, which helped to simplify the FE human model adjustment process; Lalwala et al. [44] reconstructed real-world car-to-pedestrian impact accidents using a full-scale THUMS FE model, with the purpose of investigating the kinematics behavior and evaluating the biofidelity of this model; Li et al. [45] improved the computational efficiency of the THUMS model by increasing the skeleton elements size and simplifying the lower limbs of the model, and this simplified FE pedestrian model performed well in the prediction of the pedestrian kinematics when simulating existing cadaver tests. Chai et al. [46] constructed a pedestrian model (knockdown human model) by combining a simplified FE head model (including just cortical bone, cancellous bone, encephalon and scalp) with the TNO model, connected by a dynamic hinge. However, these attempts still cannot reach the balance between accurately predicting the pedestrian brain injury and the computational efficiency.

The purpose of this paper is to utilize the existing coupling technique [46,47] to establish a new coupled pedestrian computational biomechanics model (CPCBM), capable of balancing the accuracy and computational efficiency. The head–neck part and the rest of the CPCBM are taken from THUMS (Version 4.01) FE and TNO (Version 7.5) MB 50th percentile adult male pedestrian models; each part being connected by the coupling technique [46,47]. The reliability of the CPCBM is evaluated by reconstructing three cadaver tests published in the literature [17,48,49]. This study can provide a more efficient and reliable tool for the research of pedestrian head-injury biomechanics and prevention of injury due to car impact.

## 2. Materials and Methods

### 2.1. Development of CPCBM

#### 2.1.1. Extraction of THUMS and TNO Pedestrian Models

##### Extraction of THUMS Pedestrian Model

**Figure 1:**
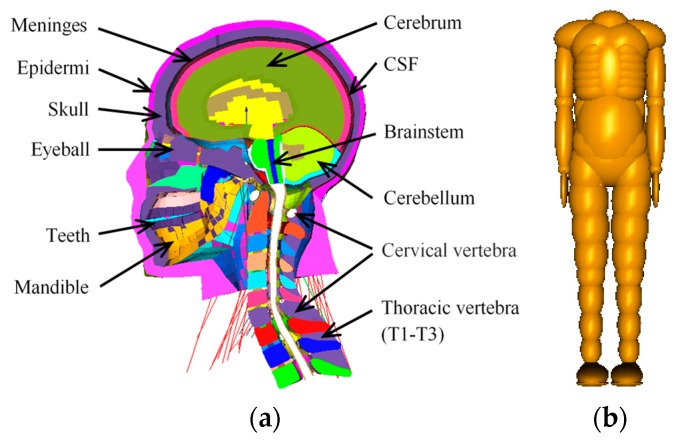
(**a**) Extracted head–neck finite element (FE) model; (**b**) extracted pedestrian multibody model.

The THUMS (Version 4.01) FE pedestrian model [4,5] consists of approximately 0.65 million nodes and 2 million elements. The stature and weight of the model are 175 cm and 77 kg, respectively. From the full-scale THUMS model, we extracted the head, neck, some one-dimensional elements connected to the head and body, and the three thoracic vertebrae (T1–T3). The head–neck complex of the THUMS model presents a detailed human anatomy structure, as shown in Figure 1a.

##### Extraction of TNO Pedestrian Model

The remaining parts of the CPCBM are taken from the TNO 50th percentile adult male pedestrian model [3,31]. The full-scale TNO model has a stature and weight of 174 cm and 75.7 kg, respectively, and it consists of 52 rigid bodies. We removed the ellipsoids (including head and neck) above the collar of the TNO model and the remaining parts of the TNO pedestrian model for constructing the CPCBM, which includes the torso, the upper and lower limbs, as shown in Figure 1b.

#### 2.1.2. Coupling of Finite Element and Multibody Parts of the CPCBM

**Figure 2:**
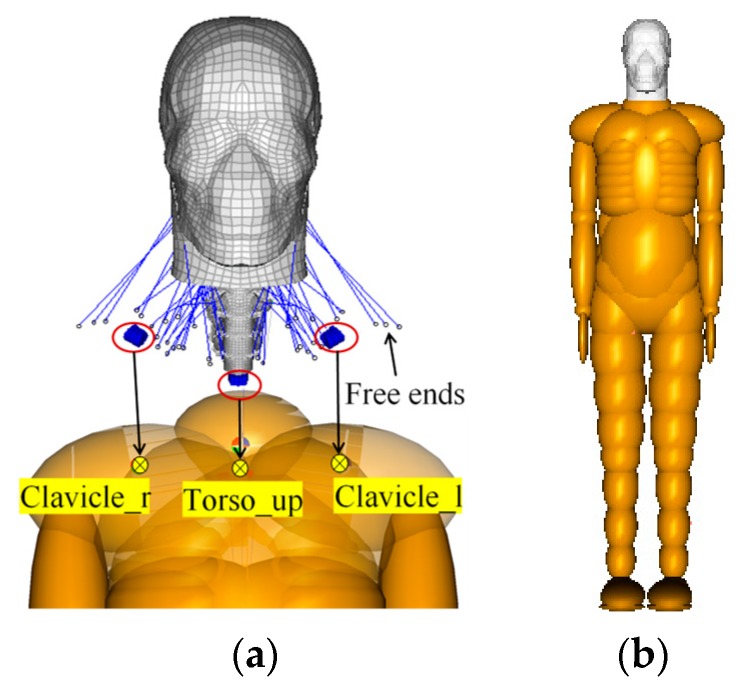
(**a**) Schematic diagram of the coupling process; (**b**) the full-scale coupled pedestrian computational biomechanics model (CPCBM).

With the head–neck complex of the THUMS model and the remaining body parts of the TNO model (see Section 2.1.1), the full-scale CPCBM can be built in the coupling assistant module available in MADYMO software package [47]. It should be noted that some auxiliary processing on the FE head–neck model before the coupling procedure needs to be performed so that the FE parts can be reasonably connected with the MB parts. For example, in the isolated head–neck model, one end of the FE one-dimensional elements (such as the line-shaped elements around the neck, Figure 2a, representing the neck muscles) that are originally connected to the FE upper torso and shoulder become free ends. These free ends cannot be directly connected to the rigid bodies (clavicle r body, torso up body and clavicle l body), so the block elements are created (the blue elements in the red circles in Figure 2a) and free ends (they are divided into three groups depending on their locations relative to the block elements) are connected to the three block elements via rigid connection. Direct connection between the block elements and the upper torso and shoulder bodies (see the markers “
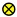
” in Figure 2a, including the upper torso and two clavicles) via the “Constraints MADYMO Body” keyword in the coupling assistant module in the MADYMO software package are created to implement the coupling of the FE head–neck complex with the remaining MB segments of the TNO pedestrian model. The coupling procedure is shown in Figure 2a, and the full-scale CPCBM is shown in Figure 2b.

### 2.2. Validation of the CPCBM

#### 2.2.1. Description of Cadaver Tests

Three cadaver impact tests from the literature were selected for the validation of the CPCBM [17,48,49]; the details of the PMHSs are listed in Table 1. The subjects were initially positioned in a nominal mid-gait stance with the left foot forward and right foot behind, and were oriented so that they were struck laterally on the subject’s right side. The arms were positioned with the hands crossed in front of the abdomen and bound at the wrist with the cable ties (for test V2374, the upper extremities were bound together at the point of amputation). For each subject, the initial position was adjusted following the positioning guidelines described in SAE J2782. During the test, the impact buck was accelerated to a target impact velocity of approximate 40 km/h, at which it contacted the pedestrian [17].

According to Wu et al. [17], a large amount of motion information from the head to the lower extremities of the subjects was recorded from the tests, including various anatomical positions (head, thoracic T1 and T8, pelvis, left and right femur, etc.). Since the attention of our study focused on the head responses of the CPCBM, only the subject’s head-related experimental data (including trajectory, displacement, velocity, acceleration) was used for the validation work.

#### 2.2.2. Modelling of the Impact Buck

The impact buck utilized in the cadaver tests was based on the sedan-type vehicle, which demonstrated a good pedestrian protection rating [50]. By using MADYMO software, an MB impact buck was built according to the detailed dimensions and the stiffness curves of the buck components described in the literature [50]. In order to simulate the impact between the FE head of the CPCBM and the windshield of the buck, we added an FE buck windshield to the buck model; the stress–strain curve of this FE windshield model was also from the literature [50]. Similarly, the FE windshield was also coupled with the MB impact buck in the same way the finite elements of the head–neck FE model and the rigid bodies of the MB model (of the remaining segments of the pedestrian) was connected during the coupling procedure of the CPCBM pedestrian model (see Section 2.1.2). The details of the impact buck model can be seen in Appendix A.

#### 2.2.3. Setting Up the Impact Simulation Between the Buck and PMHSs

##### Model Scaling and the Initial Posture Adjustment

**Figure 3:**
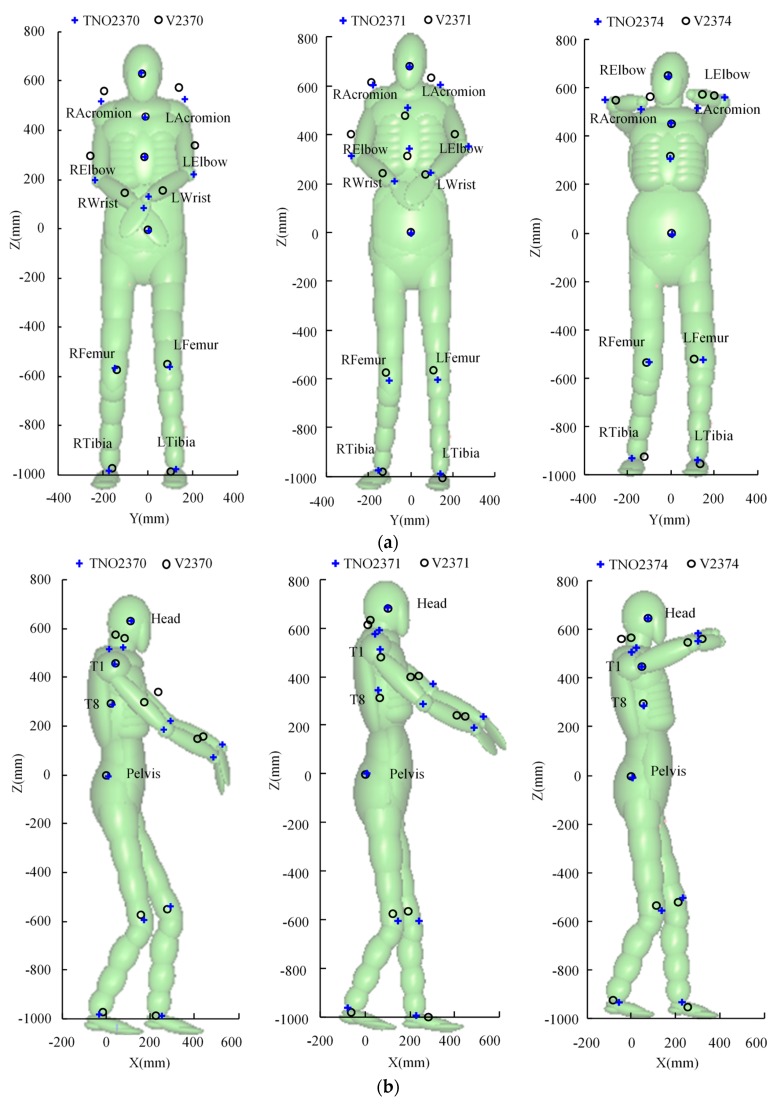
Postures over-plotted with Vicon marker positions from the experiments (aligned with the pelvis position with the model in the background) in case V2370, case V2370, and case V2374 [17]: (**a**) Y-Z plan; (**b**) X-Z plan.

The subjects’ biomechanical responses were found to be substantially influenced by their pre-impact posture and anthropometry [15,51]. In order to reduce the anthropometric differences between the CPCBM and the subjects, we scaled the TNO 50th percentile adult male pedestrian model to match the subjects in the tests, by defining detailed anthropometric parameters in the MADYSCALE module available in the MADYMO software package [31]. These signals are derived from the actual measurement data of the three subjects in the tests (see Table 2). Although the scaled TNO models are already similar to the tested subjects, some minor differences still exist between the scaled models and subjects. For example, the shoulder height of all the scaled TNO models is about 20 ± 5 mm smaller than those of the subjects, and the crotch width of the scaled models is about 25 ± 5 mm smaller than those of the subjects. Therefore, according to the actual anthropometric parameters, we appropriately double-adjusted the scaled TNO models. Afterward, following the reported information about the pre-crash posture of the subjects [49], the scaled TNO models were positioned by comparing the marking points between the scaled TNO models and the subjects (Figure 3). Finally, the subject-specific full-scale CPCBMs were created based on the scaled TNO models, following the coupling procedure in Section 2.1.1 and Section 2.1.2.

##### Buck–Pedestrian Impact Modelling

**Figure 4:**
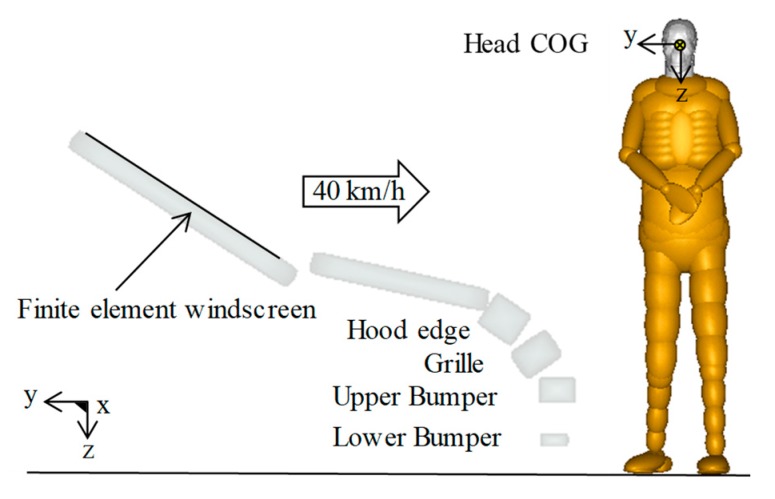
Initial condition of the buck–-pedestrian impact simulation.

In simulations, the global positions and the coordinate systems of the head model COG were set to be consistent with those of the subjects at the pre-collision stage. The motion direction of the impact buck as well as the positions and orientation of the coordinate systems in the simulation are shown in Figure 4. Similar to the tests, the simulation duration is 200 ms, starting from when the lower extremity of the CPCBM initially contacts the front of the impact buck. Following the previous study [2,17], the friction coefficients between the human model and the impact buck, and between the human model and the road surface are set to 0.3 and 0.6, respectively.

During the simulation using the CPCBM, both of the LS-DYNA solver (SMP971, LSTC, Livermore, CA, USA) and the MADYMO workplace (Version 7.5, TASS BV, Helmond, The Nether lands) were invoked by the coupling assistant module from the MADYMO software package, and the FE head–neck model and MB model (of the remaining segments of the pedestrian) were executed simultaneously using the LS-DYNA FE code and MADYMO MB code.

#### 2.2.4. Objective Rating Tool Correlation and Analysis (CORA)

Here, the objective rating tool correlation and analysis CORA [52] was used to quantitatively evaluate the correlation of the analyzed head response signals between the predictions of both the adjusted TNO model and the CPCBM, and tested PMHSs [17]; such a method has been used in similar studies [53,54]. The objective rating tool CORA uses two independent sub-ratings: a corridor rating and cross-correlation rating to assess the correlation of two signals. The rating score was calculated by taking a weighted average of these two independent sub-ratings. This rating method outputs a score ranging from 0 (no correlation) to 1 (perfect match).

## 3. Results

### 3.1. Comparison of Kinematic Response

**Figure 5:**
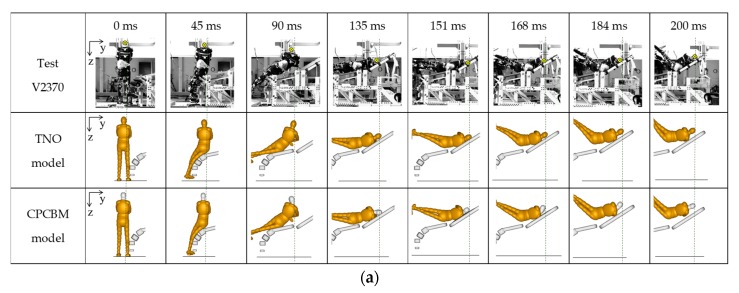

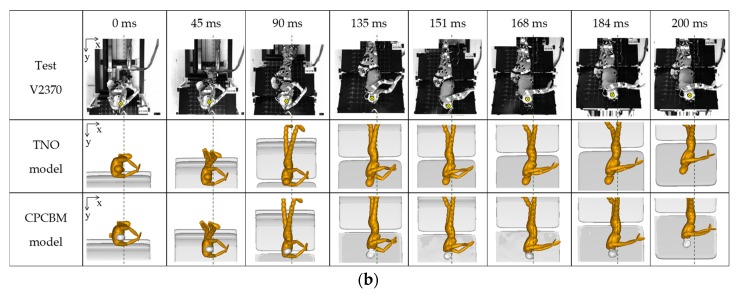
Overall kinematics comparison for test V2370 [17] between the TNO model, CPCBM, and the experimental data: (**a**) posterior view, (**b**) superior view.

The comparisons of the pedestrian’s overall motion between the simulation and the experimental measurements are shown in Figure 5, with the test V2370 as an example. It appears that the predicted kinematics using both the TNO model and CPCBM match well with the experimental results. The similar comparisons of tests V2371 and V2374 can be found in Appendix B.

**Figure 6:**
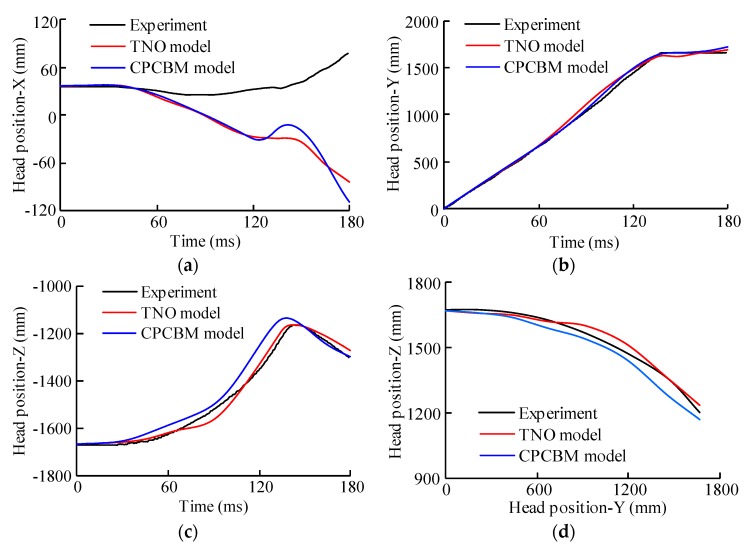

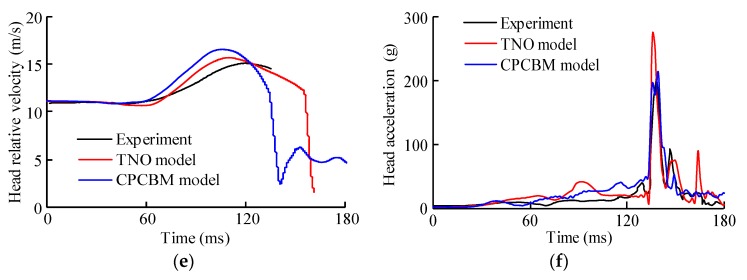
Comparisons of analyzed responses for test V2370 [17] between the TNO model, CPCBM and the experimental data: (**a**) the head center of gravity (COG) displacement in X direction, (**b**) the head COG displacement in Y direction, (**c**) the head COG displacement in Z direction, (**d**) the head COG trajectory in the YOZ plane, (**e**) The head COG resultant velocity relative to the impact buck, (**f**) the head COG resultant acceleration.

The trajectory, displacement, and resultant velocity relative to the impact buck, and resultant acceleration time histories of the head COG, calculated with the two pedestrian models (TNO and CPCBM) were also compared with the experimental results, taking the test V2370 as an example again, as shown in Figure 6. From the comparison of the displacement (Figure 6a–c), we can see that in Y/Z directions both the TNO model and CPCBM well-predict the motion of the subject’s head COG observed in the experiment, which can also be seen in the comparison of the head COG’s trajectory in the YOZ plane (Figure 6d). In terms of the resultant velocity relative to the sedan buck (Figure 6e), the difference of head impact velocity between the experiment and simulation is small, particularly for tests V2370 and V2371 (see Appendix C). A similar phenomenon can be observed in the resultant acceleration of the pedestrian head COG (see Figure 6f). Similar comparisons for tests V2371 and V2374 are shown in Appendix C.

**Figure 7:**
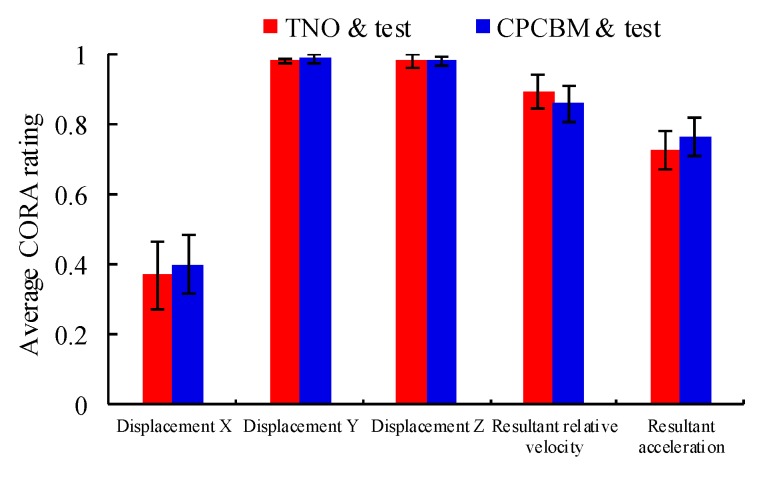
Average correlation and analysis (CORA) ratings of the correlations between the experimental results and the simulations using the TNO model and CPCBM, for all the analyzed signals.

The correlation scores between the predictions using the TNO pedestrian model and CPCBM, and the experimental results were analyzed via the CORA method, shown in Table 3. As can be seen the TNO model and CPCBM both poorly predicted the head X direction displacement of each subject in the test. Especially, for test V2374, the S_cora_ between the simulation and experiment in X-direction displacement are the worst. But we found that the analyzed correlations between the CPCBM and the test regarding the head displacement in Y and Z direction (CORA ratings: Y = 0.99 ± 0.01; Z = 0.98 ± 0.01), the resultant velocity relative to the sedan buck (CORA ratings: 0.85 ± 0.05), and the resultant acceleration (CORA ratings: 0.77 ± 0.06), are acceptable. Besides, the result of CORA ratings between the TNO model and the test are very close to the ratings of the CPCBM (Table 3 and Figure 7).

### 3.2. Comparison of Injury Prediction

**Figure 8:**
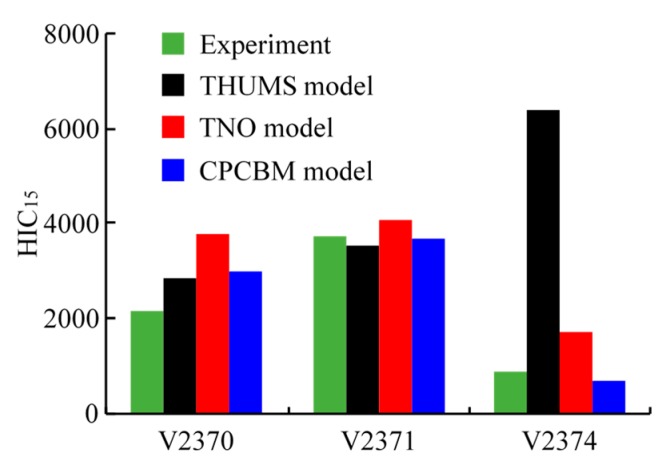
Head injury criterion 15 (HIC_15_) recorded in the experiment and obtained from different human models in the simulation.

For the experimental data on the head injury, only head injury criterion HIC_15_ was reported in the literature [17]. Thus, we also compared the HIC_15_ of PMHSs with those of the TNO model and CPCBM from the simulations (Figure 6). In addition, we also introduced the predicted HIC_15_ by the full-scale THUMS FE pedestrian model used by Wu et al. [17] as a reference (Figure 8). The predicted HIC_15_ of TNO model in three tests are all close to each subject suffered. However, the predicted HIC_15_ of the CPCBM in test V2371 is slightly smaller than the subject suffered in experiment, which has closely associated with the difference of its initial posture and anthropometric parameters. The THUMS model predicted HIC_15_ are closer with the subject in tests V2370 and V2371, but there is also a big difference in test V2374.

### 3.3. Calculation Efficiency of the Human Body Models

**Figure 9:**
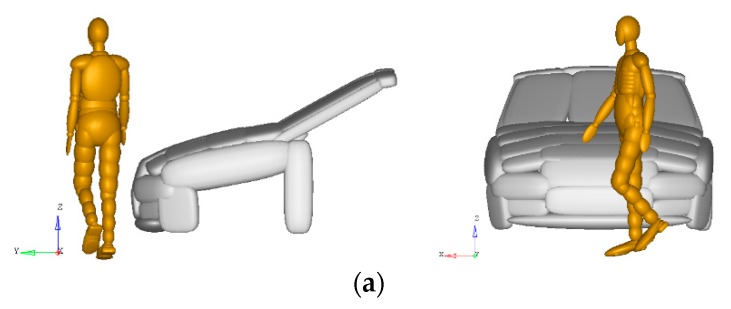

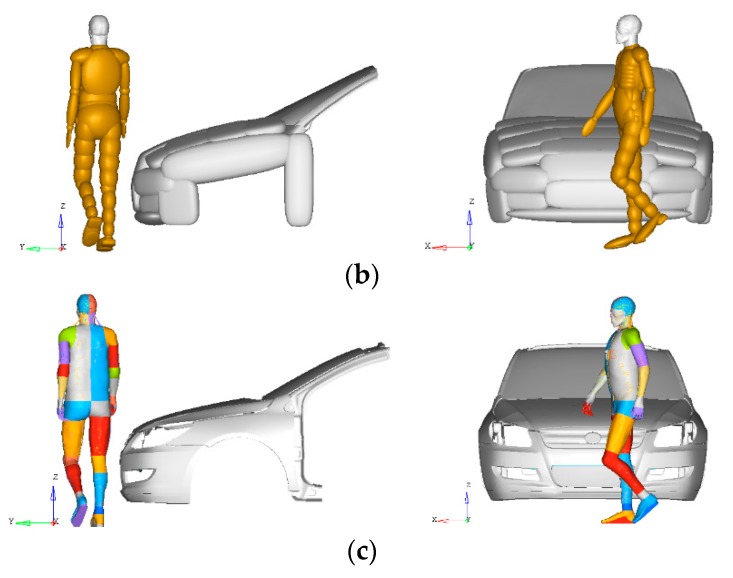
Initial conditions of car-to-pedestrian impact scenarios: (**a**) full-scale TNO multibody (MB) pedestrian model, (**b**) CPCBM, (**c**) full-scale Total Human Model for Safety (THUMS) finite element (FE) pedestrian model.

The calculation efficiency of a human body computational biomechanics model in predicting human injury was rarely mentioned in the literature. In our study, the time efficiency of the currently used two pedestrian models (the full-scale MB TNO pedestrian model and the CPCBM) were compared and evaluated by simulating the same typical car-to-pedestrian impact scenario (Figure 9) at 40 km/h lasting 240 ms using a PC with 8-core i7 CPU (Intel Core). Since the time efficiency disadvantage of the full-scale FE model was well-known [25], the THUMS pedestrian model was also included in the comparison (see Table 4). In order to ensure the comparability of the results of the models, the mass scaling percentage of all FE models were limited to 2%, and hourglass control algorithms were used to keep the ratio of hourglass energy to the total energy less than 10%, as per the recommendations of Yang and King [55]. From the comparison shown in Table 4, it can be seen that the TNO model shows extremely high computational efficiency, and the full-scale FE model requires significantly more computation time than the others. It is not surprising that the CPCBM takes far less time than the THUMS model. In addition, it is worth mentioning that the CPCBM takes substantially less time for model scaling and repositioning than the THUMS model, as the torso and upper/lower extremities of the CPCBM model were taken from the MB pedestrian model.

## 4. Discussion

### 4.1. Model Scaling and the Initial Posture Adjustment

The anthropometric parameters and initial posture of the pedestrian have significant influence on the predicted head kinematics and injury response [15,51], so the pedestrian model scaling and initial posture adjustment are important for accurately reproducing head responses in the simulation. In this study, the full-scale TNO pedestrian model was firstly scaled and adjusted following the anthropometry and initial posture of the tested PMHSs, and then used to form the CPCBM with the combination of FE THUMS head–neck complex. Although the subjects’ main measurement signals are available [49], some of the necessary parameters needed for scaling are still missing (see Table 2). In order to complete the scaling, these missing parameters were taken from the TNO 50th percentile adult male pedestrian model, which is believed to contribute to the differences between the adjusted TNO models and the marked points of the subjects (Figure 3), and may also cause a potential difference of the predicted head motion by the scaled TNO models from the experimental data. Another possible explanation of such a differences (Figure 3), may rely on the fact that, since the TNO model is a simplified MB pedestrian model, which means the outer surfaces of all the bodies are composed of rigid ellipsoid, it is quite difficult to accurately find the points of the exact same locations as the marker points of the corresponding subjects (see Figure 3).

### 4.2. Comparisons of the Dynamics Responses

In the simulations of all the chosen PHMS tests, the overall motion responses of the CPCBM and TNO models match well with the experimental results, however, it is obvious that both the CPCBM and TNO models rebound off the bonnet of the impact buck earlier than the subjects. The separate times are basically the same: it can be found from the still captures of the motion responses (Figure 5 and Appendix B) that the MB pedestrian separate with the bonnet at 135 ms, 138 ms, 141 ms when simulating tests V2370, V2371, and V2374, respectively, while in experiments, the upper torso of subjects keep in contact with the impact buck during the impact.

**Figure A8:**
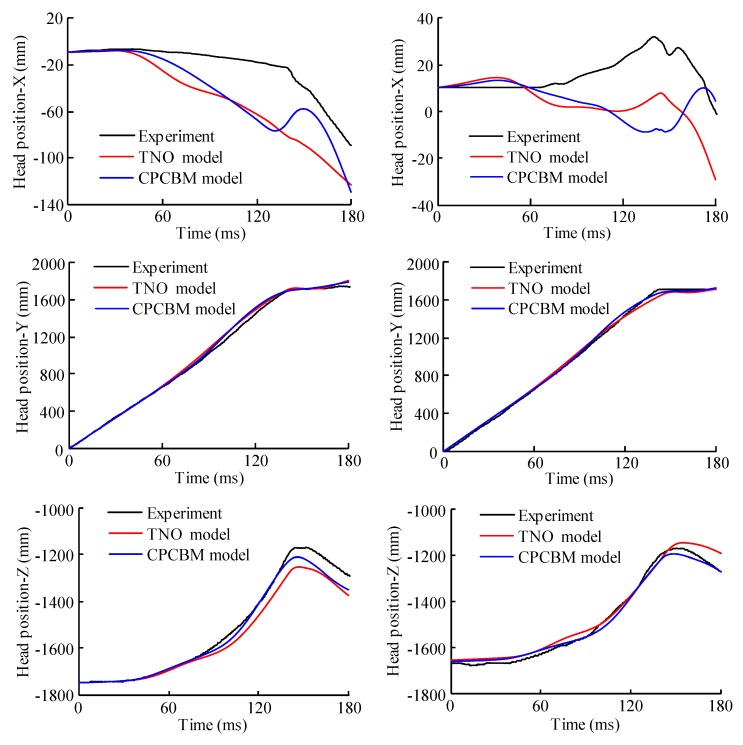
The displacement of the head COG in the X/Y/Z directions for test V2371 (left) and test V2374 (right).

Both the CPCBM and TNO models predicted well the head motions of the tested subjects, the head COG trajectories in the YOZ plane predicted using the CPCBM and TNO model are basically consistent with the experimental results (see Figure 6 and Appendix C Figure A8). However, the differences in the displacement of the head COG were observed between the predictions of the CPCBM and TNO models and the experimental results in the X direction (see Figure 6a), which was also reflected by the observation of the correlation evaluation CORA scores in Table 3. Specifically, the correlation evaluation score S_cora_ between the predictions of TNO model and CPCBM and the experimental results for test V2374 are 0.244 and 0.297, respectively, and for tests V2370 and V2371, the scores are slightly higher (see Table 3). The differences may be caused by three factors: first, there are slight differences in the anthropometric parameters and the initial posture between the subject-specific TNO pedestrian models and tested subjects, as described above; secondly, the TNO pedestrian model has a limited biofidelity; lastly, as shown in the Table A1 in Appendix D, there are clear differences between head–neck systems from the TNO model and the THUMS model. The moment of inertia of the head–neck systems scaled from the TNO model according to the PMHSs tested in three chosen experiments are larger than the THUMS head–neck model. These differences may potentially influence the head kinematics of the pedestrian models.

Though both CPCBM and TNO models showed difference in overall motion and head kinematics responses from the experimental results, the predictions of the two models for all the subjects are quite close to each other (see Table 3, Figure 7, and Appendix C). Such similarity could be largely expected since the main body regions except the head–neck complex of the CPCBM and TNO model are the same. From this perspective, a particular emphasis could be paid on the observing the differences in the predicted head kinematics between the CPCBM and the TNO pedestrian model by simulating the same cadaver tests, for the evaluation of the reliability of the CPCBM. Specifically, Figure 7 shows that the CORA ratings of the CPCBM and TNO model with the experimental results for a chosen test are quite similar, and the head motion and trajectories predicted using the two models are almost the same before the head-buck windshield contact (see Figure 6); the differences occur only when the head rebounds off. Such differences possibly contribute to the differences in the neck stiffness between the MB and FE human body models. The previous studies have already identified that the FE neck model is softer than the real human neck [25].

### 4.3. Head Injury Prediction

As the available data in the literature about the cadaver head injuries was limited [17], only the HIC_15_ was analyzed for the effectiveness evaluation of the head injury prediction of the CPCBM. The comparison of HIC_15_ between the predictions using the different pedestrian models and the test data shown in Figure 8 appears to indicate that the predictions by both the CPCBM and TNO model generally agree with the experimental results, especially for tests V2371 and V2374. In addition, the HIC_15_ values of the CPCBM and TNO model are also close to the THUMS model, except for the test V2374. Overall, the CPCBM can reproduce HIC of the pedestrians used in the experiments [17].

### 4.4. Quantitative Evaluation Method CORA

In this paper, the total ratings (S_cora_) for each type of signal (that include the head’s COG’s trajectory, displacement, and resultant velocity relative to the sedan buck and resultant acceleration) were calculated in Table 3, we can more objectively evaluate the correlation between the simulated and the experimental signals. However, it should be noted that there are some shortcomings. For example, the selection of the signal interval for evaluation and the setting of the weighting factors of its two sub-ratings during the evaluation process would affect the predicted S_cora_. In the present work, we used the start and end points of each signal as the endpoints of the signal evaluation interval, and adjusted the default weighting factor to make the evaluation process stricter. The settings used in each signal evaluation process are the same, and the details of all signals’ CORA evaluation process are shown in Appendix E.

### 4.5. Limitations

There are some limitations existing in this work. Firstly, since the TNO multibody pedestrian model does not represent the detailed human anatomic structure, it is difficult to exactly find the output points corresponding to the subjects’ anatomical marking points (e.g., T1, T8, Tibia, etc.) from the TNO model. Therefore, this paper didn’t analyze the differences in human body motion response between the CPCBM and the subjects, and only analyzed the head motion response of the CPCBM. Secondly, considering that the difference in body size between the PMHSs (the medium values in Table 5) in the tests we used and the THUMS model (see Table 5) is not significant, and the specific data of the heads of the cadavers were not reported [17,48,49], we did not scale the THUMS head–neck model during the coupling procedure, which may influence the head kinematics responses of the CPCBM. Finally, the brain injury prediction of the CPCBM is not reconfirmed in this work, due to the absence of brain injury information about the PMHSs used in the tests we referenced [17,48,49], and the fact that the THUMS head–neck model has already been validated in the literature in terms of brain injury prediction [56,57,58,59]. However, further application of the CPCBM into real-world car-to-pedestrian impact accident brain injury prediction evaluation is needed in the future.

## 5. Conclusions

A new coupled pedestrian computational biomechanics model (CPCBM) was developed by coupling existing commercial FE and MB pedestrian models, and the predicted head kinematics and injury responses obtained by simulating three previously published cadaver impact tests were compared with the experimental results for the validation of the CPCBM. Based on the results of the current study, the following conclusions can be drawn:

(1) The comparative analysis and the correlation evaluation via the CORA method indicate that the CPCBM can reasonably predict the head center of gravity (COG) kinematics and injury response of the pedestrian due to car impact in the impact direction. The predicted trajectories in the impact direction (YOZ plane) strongly agree with the experimental results (CORA ratings: Y = 0.99 ± 0.01; Z = 0.98 ± 0.01); the resultant velocity relative to the impact buck correlates well with the test data (CORA ratings: 0.85 ± 0.05); however, the correlation of the acceleration is less strong (CORA ratings: 0.747 ± 0.06).
(2) The CPCBM performed quite similarly to the full-scale TNO multibody pedestrian model in predicting the head COG kinematics and injury response of the tested subjects, which means the CPCBM carried over the pedestrian kinematics prediction performance of the TNO model that has been already widely verified, despite the replacement of the head–neck complex with the FE model.
(3) Compared to the full-scale THUMS model, the CPCBM showed a remarkable advantage in the time efficiency of the scaling, posture adjustment, and impact simulation computation when used to calculate the pedestrian brain tissue level injury during a complex accident, which suggests that the CPCBM may serve as an efficient tool for pedestrian head/brain injury research due to car impact.

## Figures and Tables

**Table 1:** Anthropometries and injuries of the tested subjects.

Test No.	Age	Gender	Height (mm)	Weight (kg)	Cause of Death
V2370	73	male	1795	72.6	Heart Failure
V2371	54	male	1870	81.6	Colon Cancer
V2374 ^1^	67	male	1780	78	COPD ^2^

^1^ This subject had its distal upper extremities amputated bilaterally at the mid-forearm; ^2^ Chronic obstructive pulmonary disease.

**Table 2:** Subjects anthropometry parameters used to scale in MADYSCALE.

No.	Body Parameters	Unit	V2370	TNO2370	V2371	TNO2371	V2374	TNO2374
1	Weight	kg	72.6	72.6	81.6	81.6	78.0	78.0
2	Standing height	mm	1795.0	1795.0	1870.0	1870.0	1780.0	1780.0
3	Shoulder height ^1^	mm	1555.0	1534.2	1620.0	1611.8	1550.0	1553.2
4	Armpit height	mm	1400.0	1434.1	1470.0	1498.9	1400.0	1139.3
5	Waist height	mm	1090.0	1128.1	1123.0	1163.2	1070.0	1125.6
6	Seated height	mm	957.2	949.8	1037.2	1029.5	970.0	964.4
7	Head length	mm	185.0	185.6	205.0	195.6	198.0	181.1
8	Head breadth	mm	161.0	156.6	157.0	153.4	158.0	154.2
9	Head to chin height ^1^	mm	208.5	226.9	238.5	237.4	228.0	225.9
10	Neck circumference	mm	452.0	408.2	352.0	395.6	445.0	398.6
11	Shoulder breadth	mm	393.0	389.5	404.0	403.5	381.0	367.3
12	Chest depth	mm	234.0	243.9	208.0	218.6	225.0	249.6
13	Chest breadth	mm	321.0	316.8	330.0	341.7	293.0	298.4
14	Waist depth	mm	180.0	179.8	190.0	201.1	190.0	199.2
15	Waist breadth	mm	313.0	296.6	360.0	348.8	321.6	298.3
16	Buttock depth	mm	228.9	214.5	237.2	232.2	240.5	237.9
17	Hip breadth, standing ^1^	mm	345.5	339.0	345.5	368.4	310.0	328.8
18	Shoulder to elbow length	mm	390.0	388.2	370.0	356.2	355.0	356.7
19	Forearm-hand length	mm	487.0	463.2	500.0	488.7	451.0	446.3
20	Biceps circumference	mm	247.0	301.4	265.0	282.6	275.0	301.4
21	Elbow circumference	mm	239.0	219.8	254.0	250.6	258.0	219.8
22	Forearm circumference	mm	226.0	229.2	233.0	241.4	210.7	232.3
23	Wrist circumference	mm	165.0	148.2	175.0	167.8	165.0	144.4
24	Knee height, seated	mm	585.0	569.8	570.0	522.7	565.0	553.8
25	Thigh circumference	mm	499.0	414.5	530.0	496.1	510.0	496.1
26	Upper leg circumference	mm	395.0	376.8	362.0	401.9	415.0	414.5
27	Knee circumference	mm	349.0	332.4	380.0	376.8	386.0	345.4
28	Calf circumference	mm	334.0	314.3	305.0	332.8	327.0	364.2
29	Ankle circumference	mm	190.0	257.5	245.0	282.6	238.0	282.6
30	Ankle height, outside	mm	92.0	102.3	90.0	101.6	88.0	97.6
31	Foot breadth	mm	90.0	99.8	87.0	108.2	86.0	102.9
32	Foot length	mm	238.0	241.5	238.0	254.4	198.0	217.7
33	Hand breadth ^1^	mm	88.6	89.5	88.6	93.6	89.1	96.6
34	Hand length ^1^	mm	192.1	187.5	192.1	188.6	191.6	182.3
35	Hand depth ^1^	mm	27.4	28.1	27.4	30.4	27.6	30.3

^1^ This parameter was not given in Forman et al. [49], but it is essential for the scale process. This parameter was calculated by another scale process where only height and weight of the samples were used to scale the Netherlands Organisation for Applied Scientific Research (TNO) 50th percentile adult male pedestrian model into the target models.

**Table 3:** Detailed CORA rating scores (S_cora_) between simulations and experimental results.

Signal	Test V2370		Test V2371		Test V2374	
	TNO & Test	CPCBM & Test	TNO & Test	CPCBM & Test	TNO & Test	CPCBM & Test
Displacement-X	0.388	0.397	0.479	0.507	0.244	0.297
Displacement-Y	0.977	0.998	0.973	0.968	0.989	0.997
Displacement-Z	0.996	0.965	0.956	0.979	0.990	0.999
Resultant relative velocity	0.932	0.840	0.921	0.924	0.821	0.808
Resultant acceleration	0.739	0.759	0.780	0.825	0.651	0.717

**Table 4:** Comparison of the computation time needed by different models.

Model Type	Impact Duration	Calculation Time	Solver
Full-scale TNO model	240 ms	1 min	MADYMO
Full-scale THUMS model	240 ms	42 h	LS-DYNA
CPCBM	240 ms	7.5 h	LS-DYNA & MADYMO

**Table 5:** The comparison of anthropometry parameters between the subjects [17] we chose and the THUMS model.

Parameter	V2370	V2371	V2374	Mean ± Standard	THUMS Model
Weight (kg)	72.6	81.6	78	77.4 ± 3.7	77
Height (cm)	179.5	187.0	187.0	181.5 ± 3.9	175

**Figure A1:**
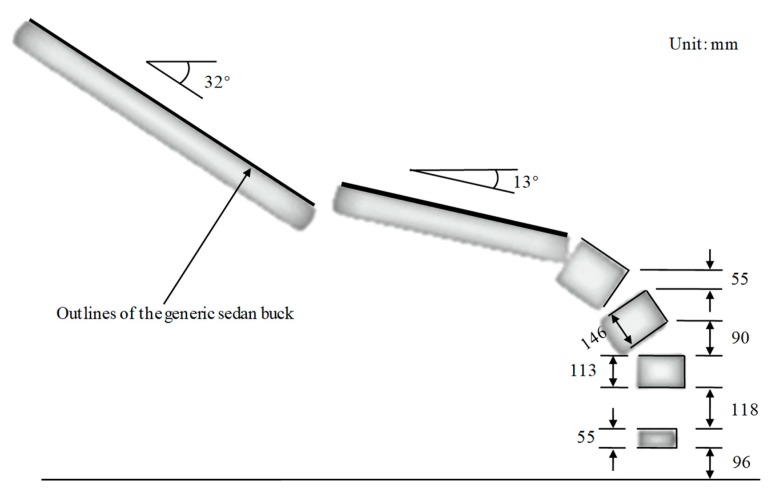
Schematic diagram of the structure of the generic sedan buck, modified from [50].

**Figure A2:**
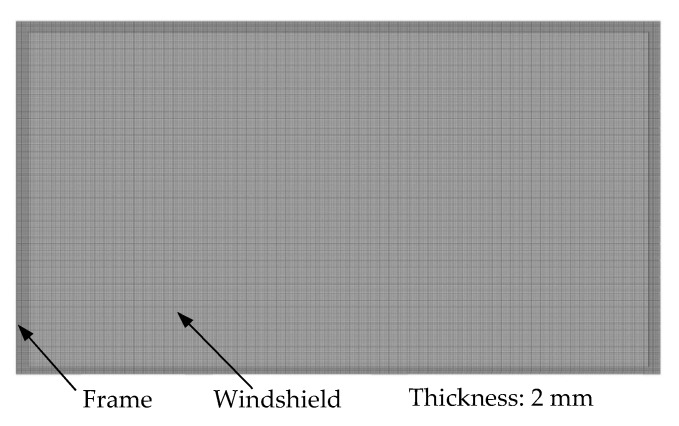
Schematic diagram of the simplified finite element windshield model.

**Figure A3:**
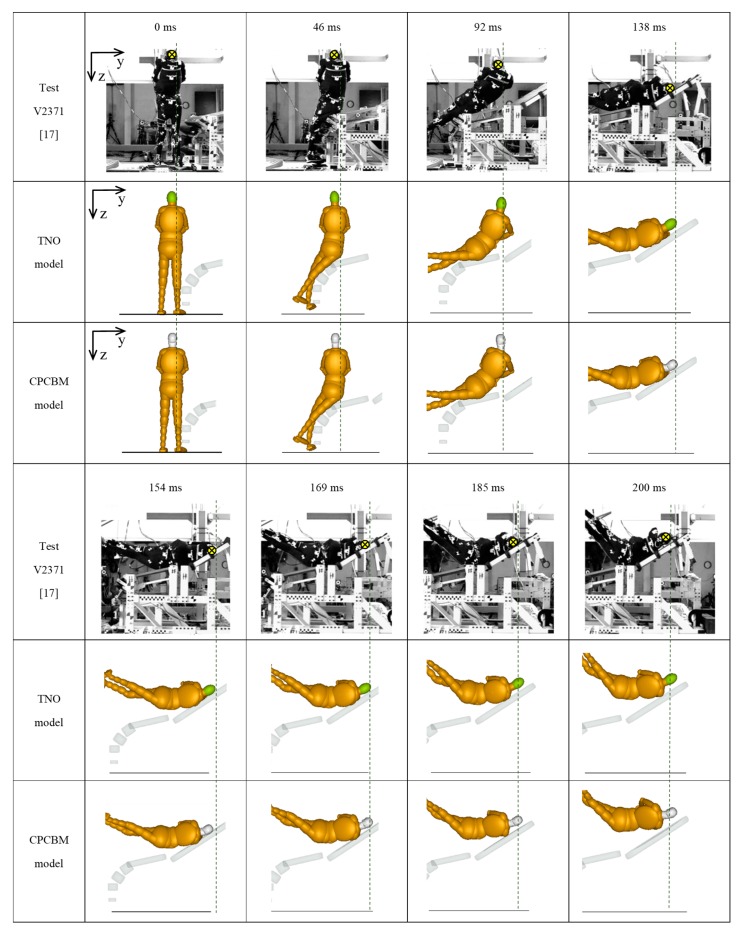
Motion responses of PMHS, the TNO model (gold color), and CPCBM (mixed color) for test V2371 (posterior view) [17].

**Figure A4:**
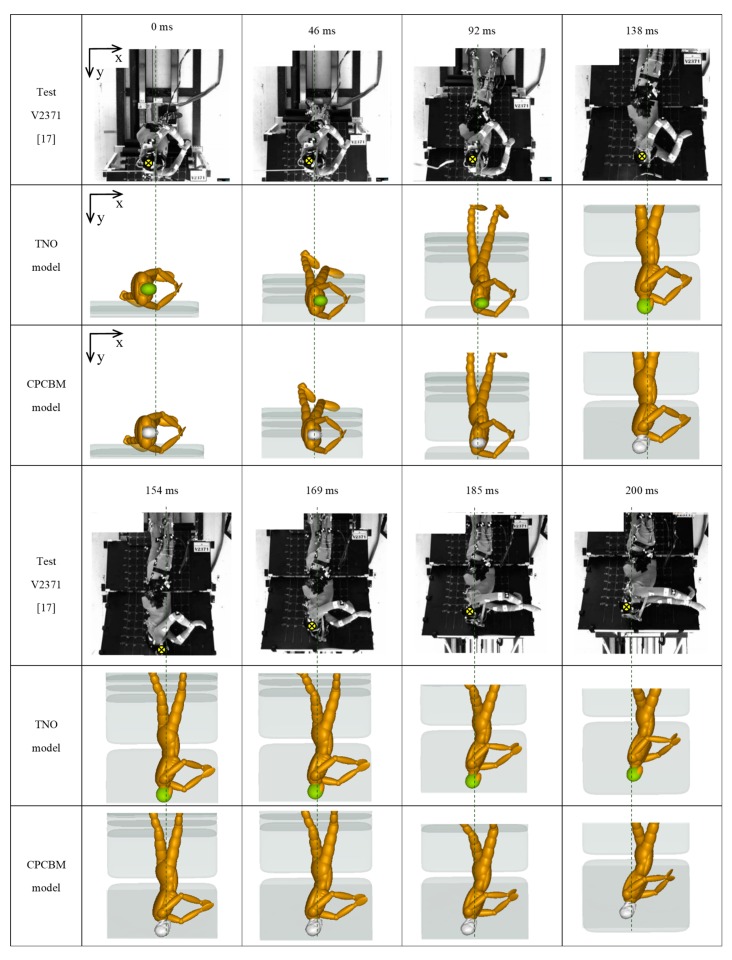
Motion responses of PMHS, the TNO model (gold color), and CPCBM (mixed color) for test V2371 (superior view) [17].

**Figure A5:**
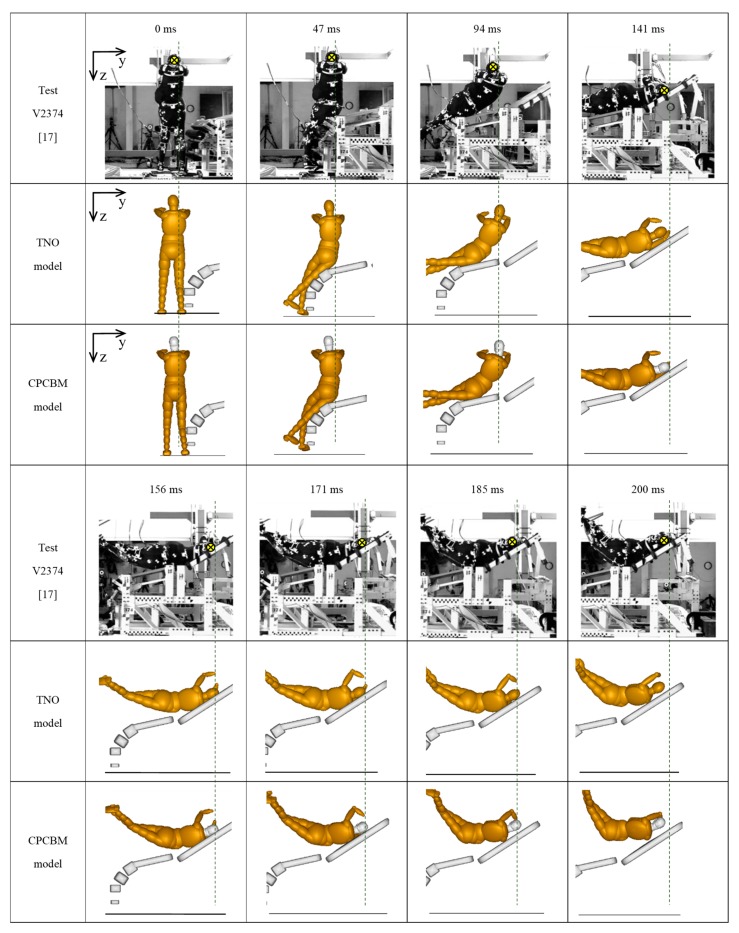
Motion responses of PMHS, the TNO model (gold color), and CPCBM (mixed color) for test V2374 (posterior view) [17].

**Figure A6:**
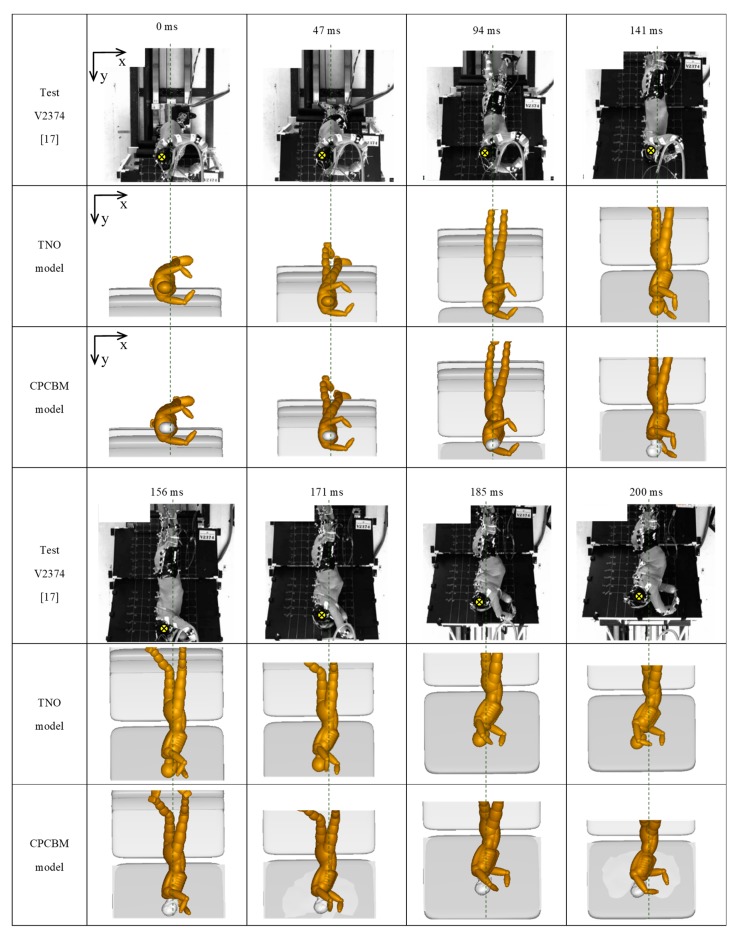
Motion responses of PMHS, the TNO model (gold color), and CPCBM (mixed color) for test V2374 (superior view) [17].

**Figure A7:**
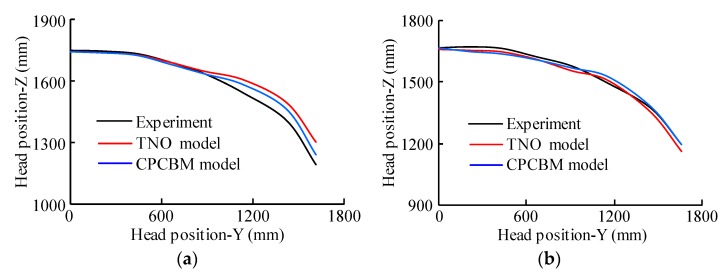
The trajectory of the head COG in the YOZ plane: (**a**) test V2371 and (**b**) test V2374.

**Figure A9:**
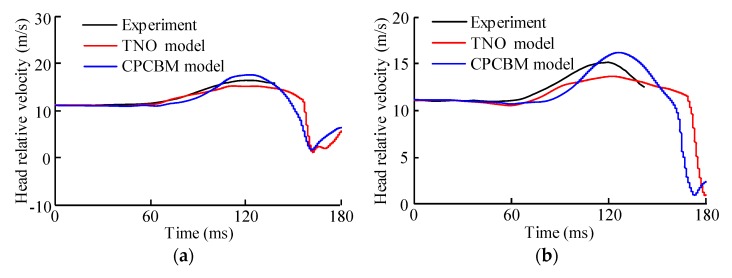
The resultant velocity of the head COG relative to the sedan buck: (**a**) test V2371; (**b**) test V2374.

**Figure A10:**
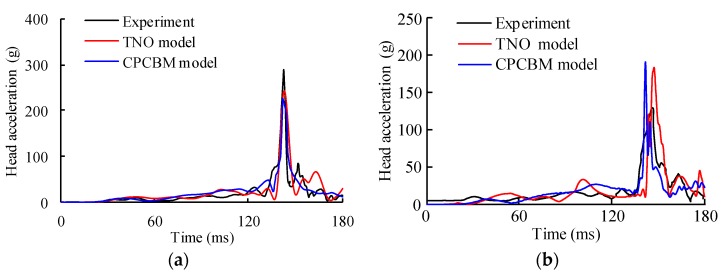
The resultant acceleration of the head COG: (**a**) test V2371; (**b**) test V2374.

**Figure A11:**
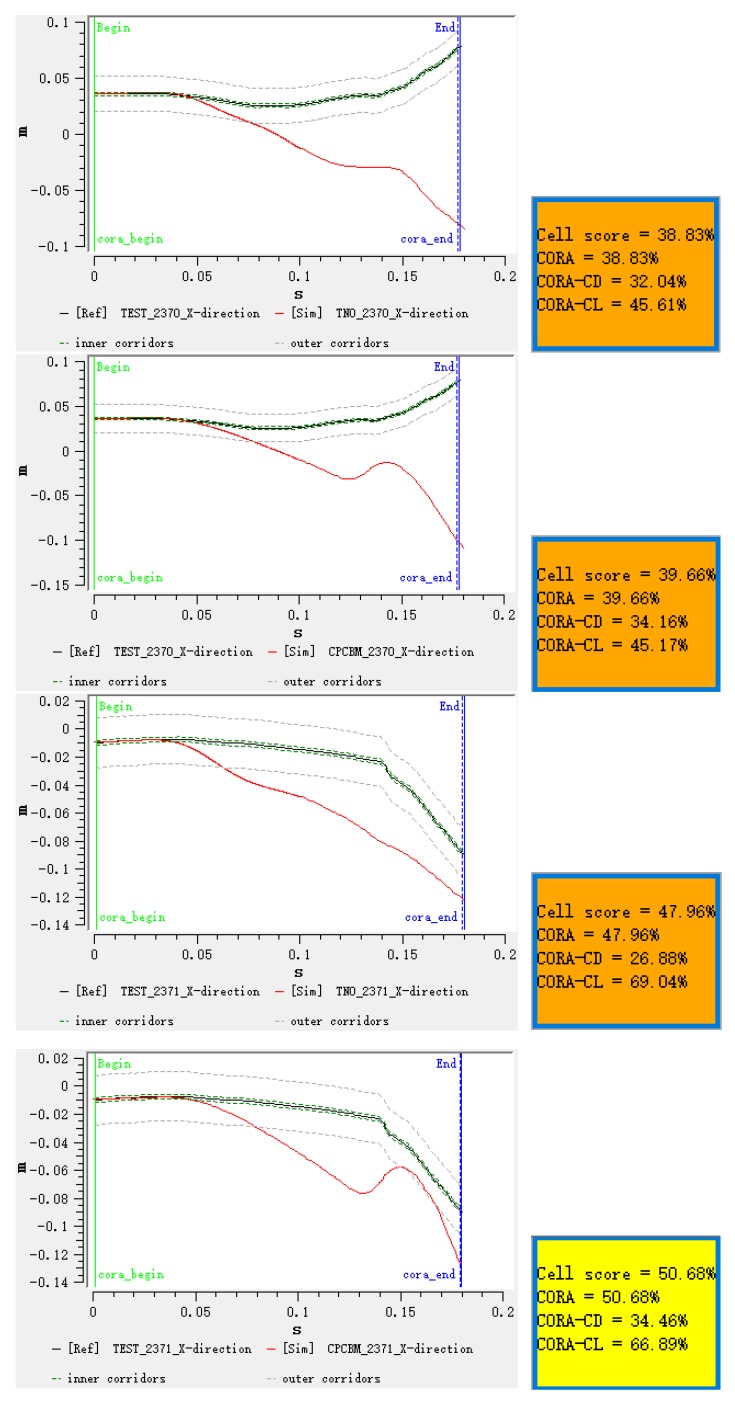

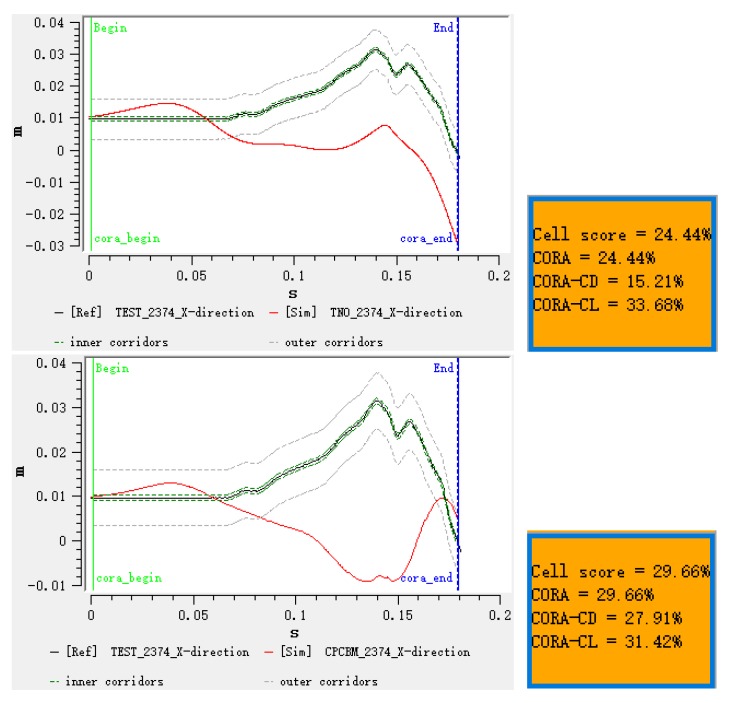
X-direction displacement signals between experiment and simulation.

**Figure A12:**
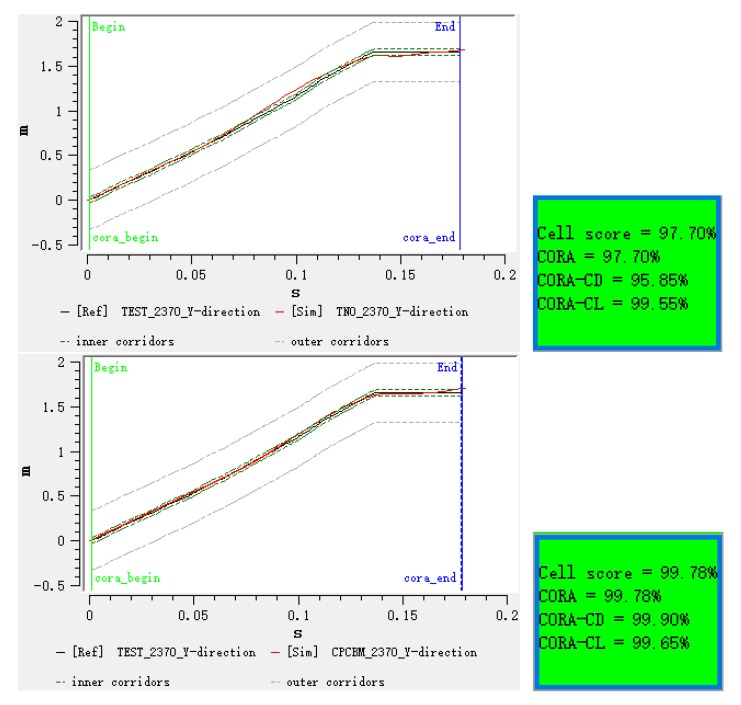

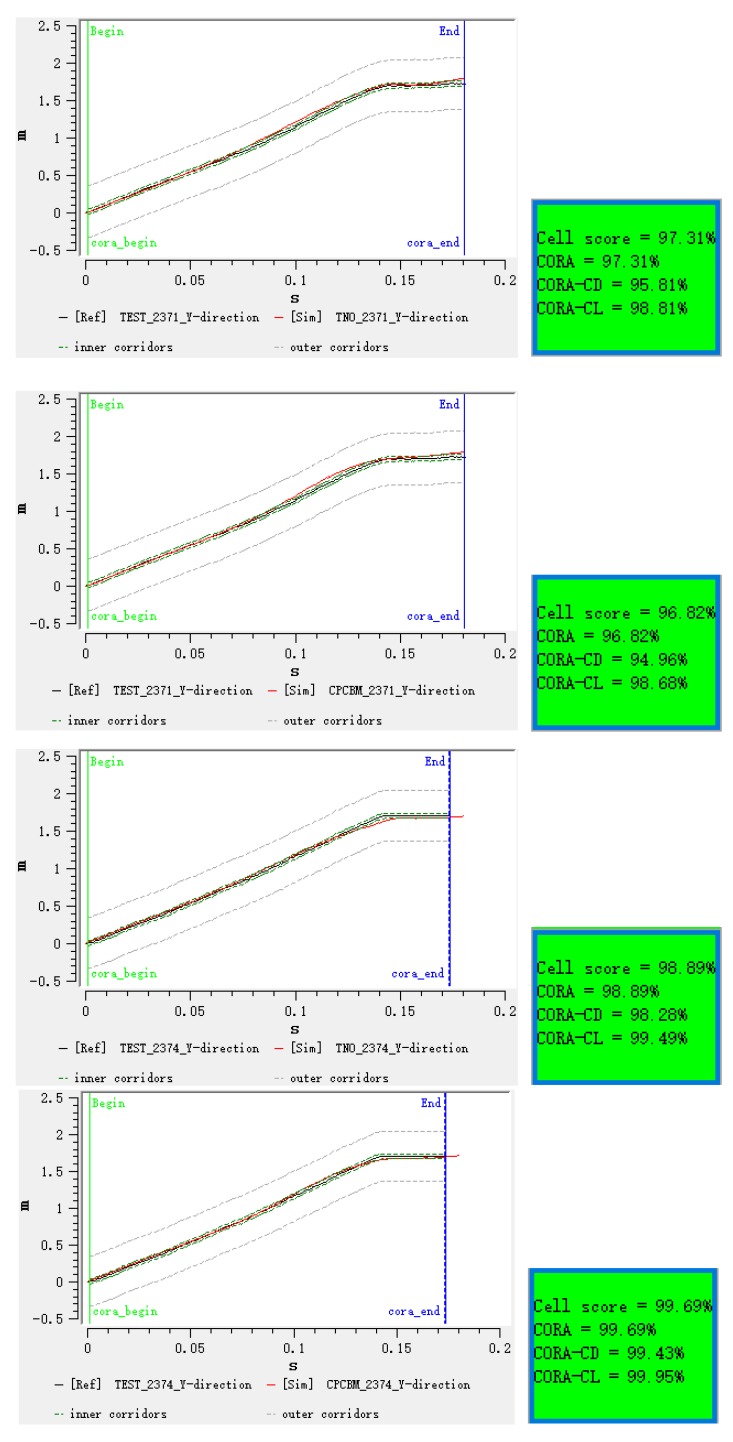
Y-direction displacement signals between experiment and simulation.

**Figure A13:**
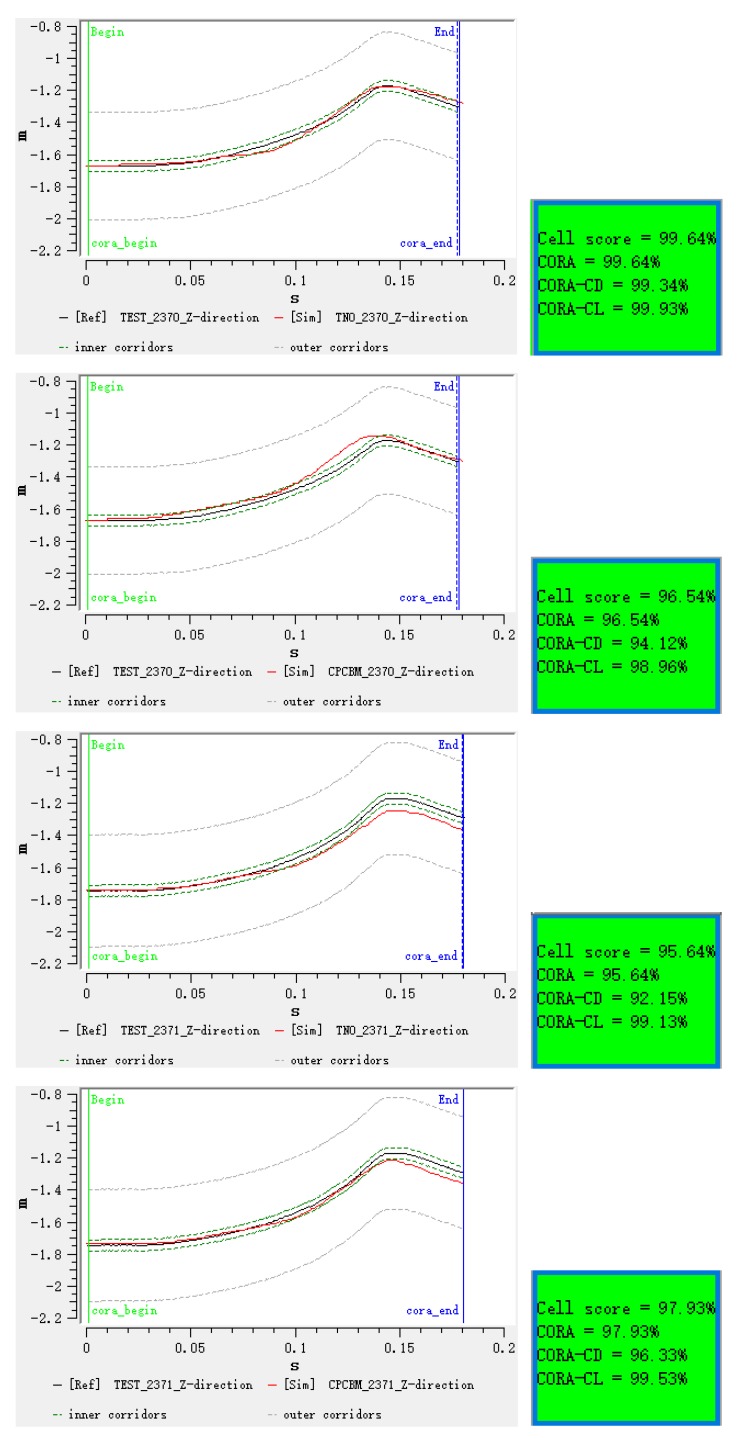

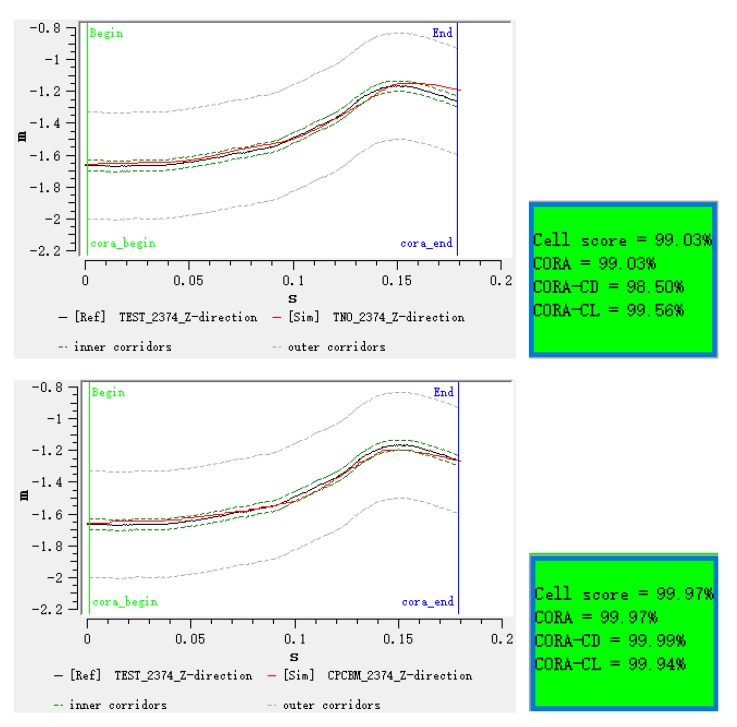
Z-direction displacement signals between experiment and simulation.

**Figure A14:**
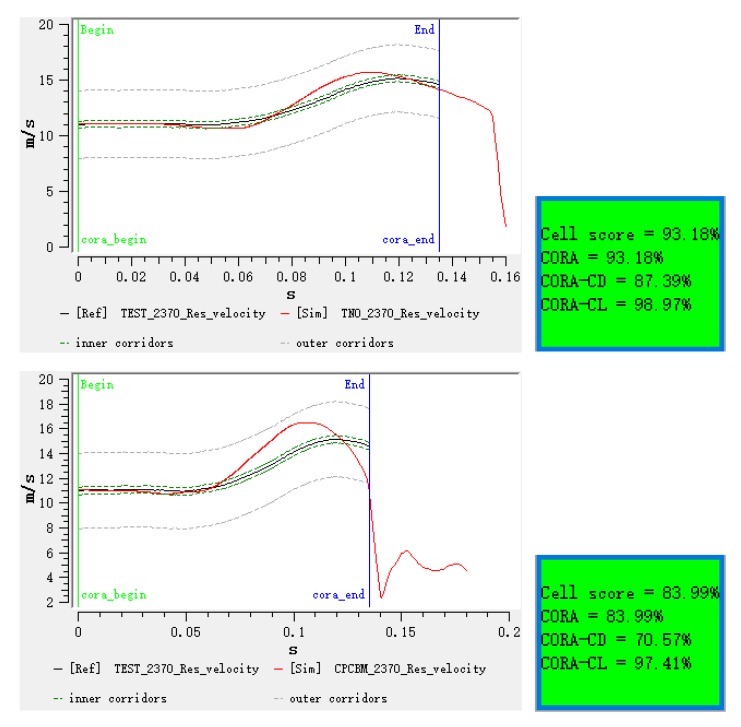

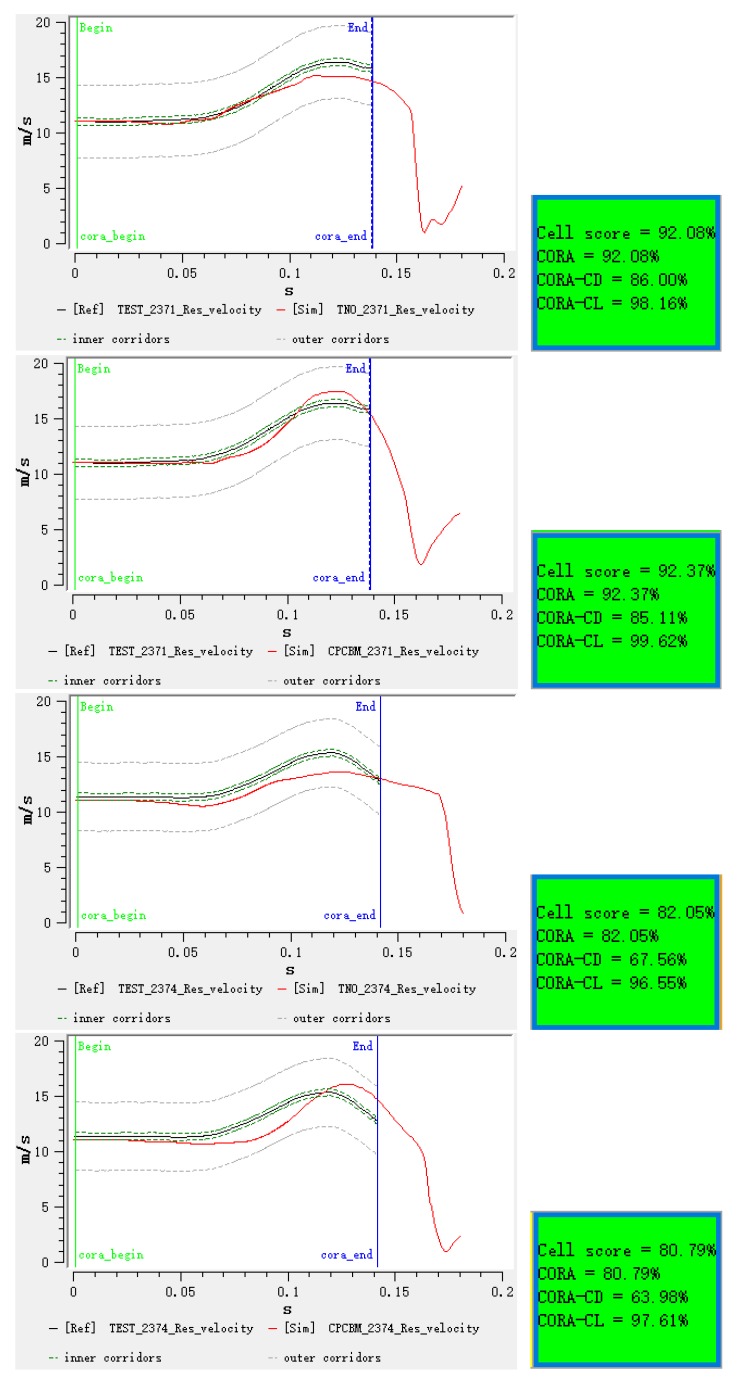
Resultant velocity signals relative to the sedan buck between experiment and simulation.

**Figure A15:**
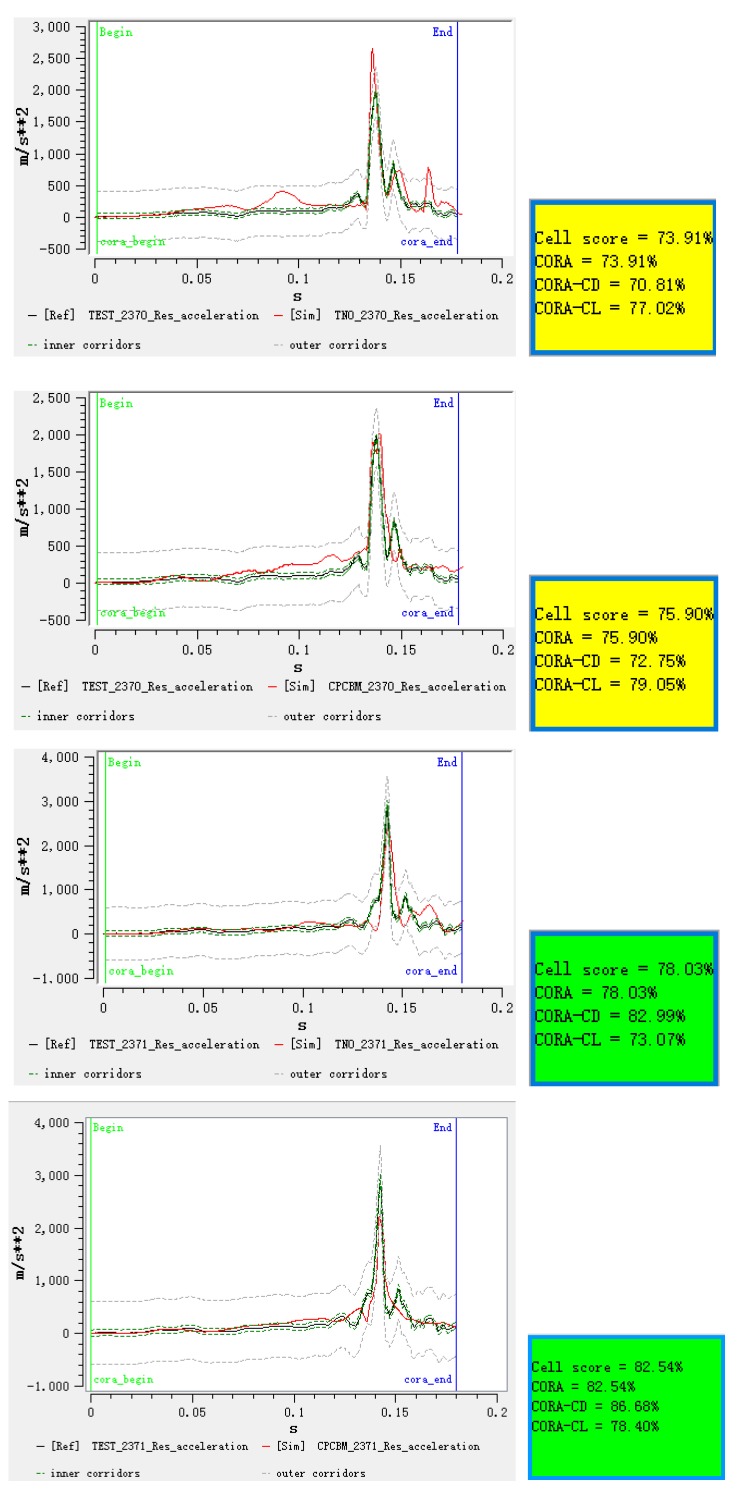

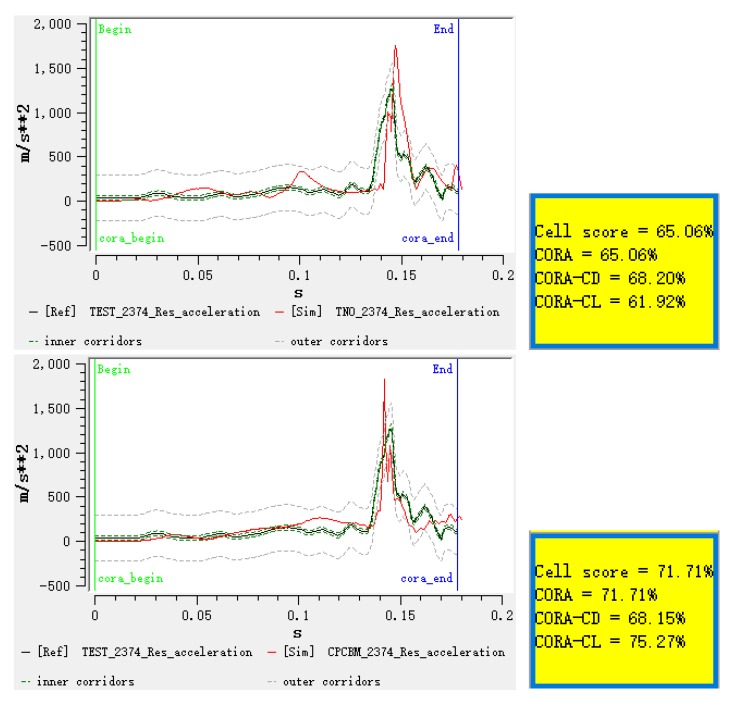
Resultant acceleration signals between experiment and simulation.

## Appendix A. Generic Sedan Buck Model

The outlines of the multibody generic sedan buck were extracted from the literature [50], in which detailed parameters and stiffness curves of the buck were mentioned. So, we can accurately reproduce the buck with rigid bodies that used in the PMHS tests (Figure A1). Additionally, a simplified finite element windshield model matched the stress–strain curve from the work is also coupled with the multibody buck. The finite element windshield model is shown in Figure A2. The multibody impact buck model used in this work includes bumper lower and bumper, grille, hood edge and hood, and windshield. The finite element windshield model consists of about 54,000 nodes and 58,000 elements, and the element size is 5 mm. The force-displacement characteristics of multibody components and material properties of the FE windshield model are all from the literature [50].

## Appendix B. Still Captures

Figure A3, Figure A4, Figure A5 and Figure A6 show still captures from the posterior and superior view. The PMHS motion captured by high-speed video cameras (grayscale) are compared to the TNO model and CPCBM respectively. In the motion comparison, the first frame is the frame of leg contact and final frame is t = 200 ms.

## Appendix D. The Comparison of Anthropometry Parameter

**Table A1:** Comparison of inertial properties of the head–neck system from the THUMS and TNO models.

Models	The THUMS Head-Neck Model	The TNO Head-Neck Model

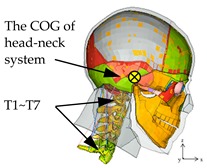
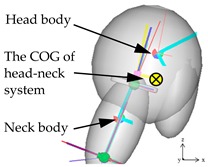
	
Total mass	5.66 kg	6.21/6.39/6.3 kg
Moment of inertia of the Head-neck system model for test V2370	I_xx_ = 1.5126 × 10^−2^ kg·m^2^ I_yy_ = 1.7991 × 10^−2^ kg·m^2^ I_zz_ = 1.0557 × 10^−2^ kg·m^2^	I_xx_ = 1.7090 × 10^−2^ kg·m^2^ I_yy_ = 1.8274 × 10^−2^ kg·m^2^ I_zz_ = 1.3113 × 10^−2^ kg·m^2^
Moment of inertia of the Head-neck system model for test V2371		I_xx_ = 2.5004 × 10^−2^ kg·m^2^ I_yy_ = 2.8844 × 10^−2^ kg·m^2^ I_zz_ = 1.7861 × 10^−2^ kg·m^2^
Moment of inertia of the Head-neck system model for test V2374		I_xx_ = 2.1877 × 10^−2^ kg·m^2^ I_yy_ = 2.4686 × 10^−2^ kg·m^2^ I_zz_ = 1.6003 × 10^−2^ kg·m^2^
